# Supplementary material for: Engineering multifunctional bactericidal nanofibers for abdominal hernia repair
Source: Commun Biol. 2021 Feb 19;4:233. doi: 10.1038/s42003-021-01758-2 (PMC7896057; doi:10.1038/s42003-021-01758-2)
Supplement: Supplementary file 2 — Supplementary Information [file 42003_2021_1758_MOESM2_ESM.pdf]

Supplementary Information

Engineering Multifunctional Bactericidal Nanofibers for Abdominal Hernia Repair

Samson Afewerki,* Nicole Bassous, Samarah Vargas Harb, Marcus Alexandre F. Corat, Sushila Maharjan, Guillermo U. Ruiz-Esparza, Mirian M. M. de Paula, Thomas J. Webster, Carla Roberta Tim, Bartolomeu Cruz Viana, Danquan Wang, Xichi Wang, Fernanda Roberta Marciano, and Anderson Oliveira Lobo*

Supplementary Figures

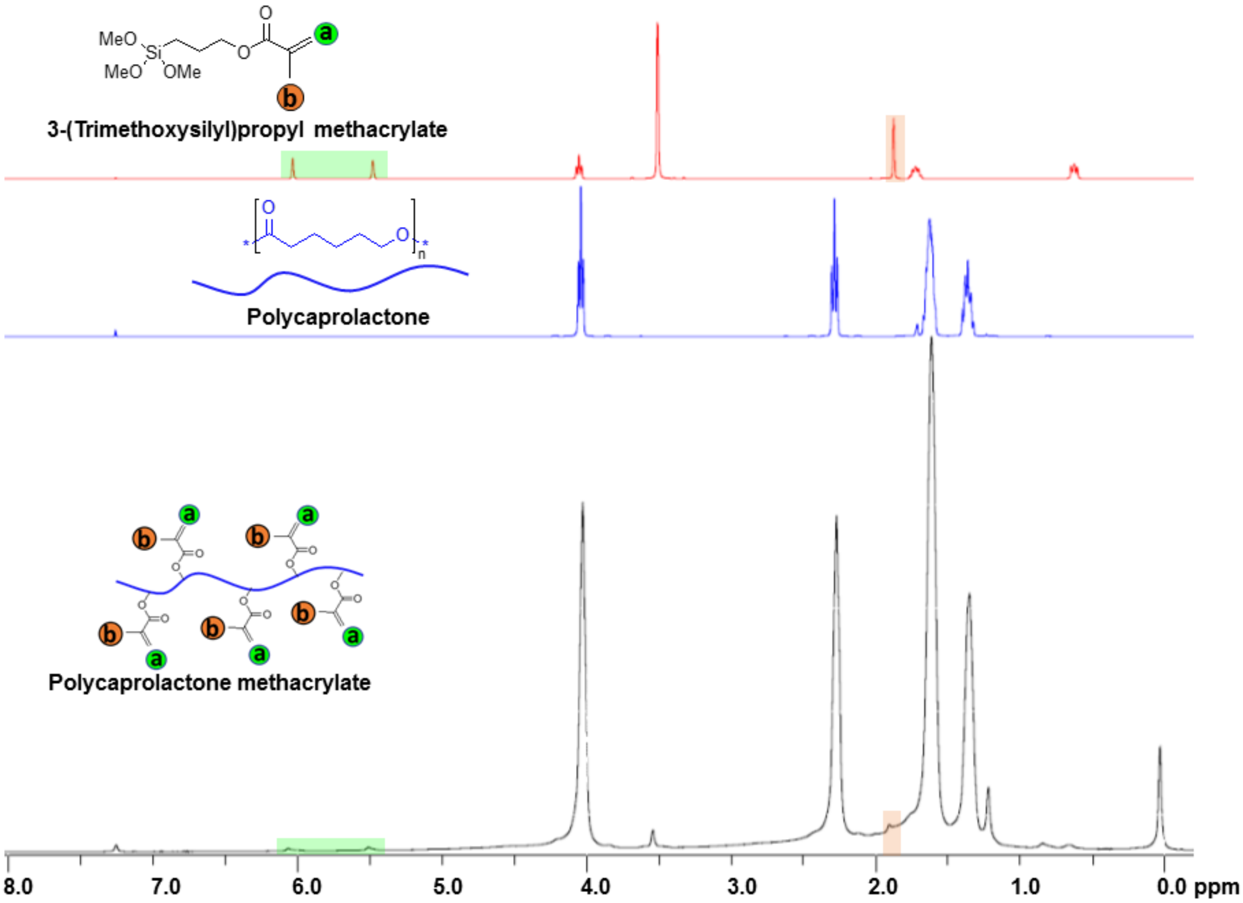

Supplementary Fig. 1 The ^{1}H NMR of 3-(Trimethoxysilyl)propyl methacrylate, polycaprolactone and polycaprolactone covalently conjugated with silane methacrylate (PCLMA).

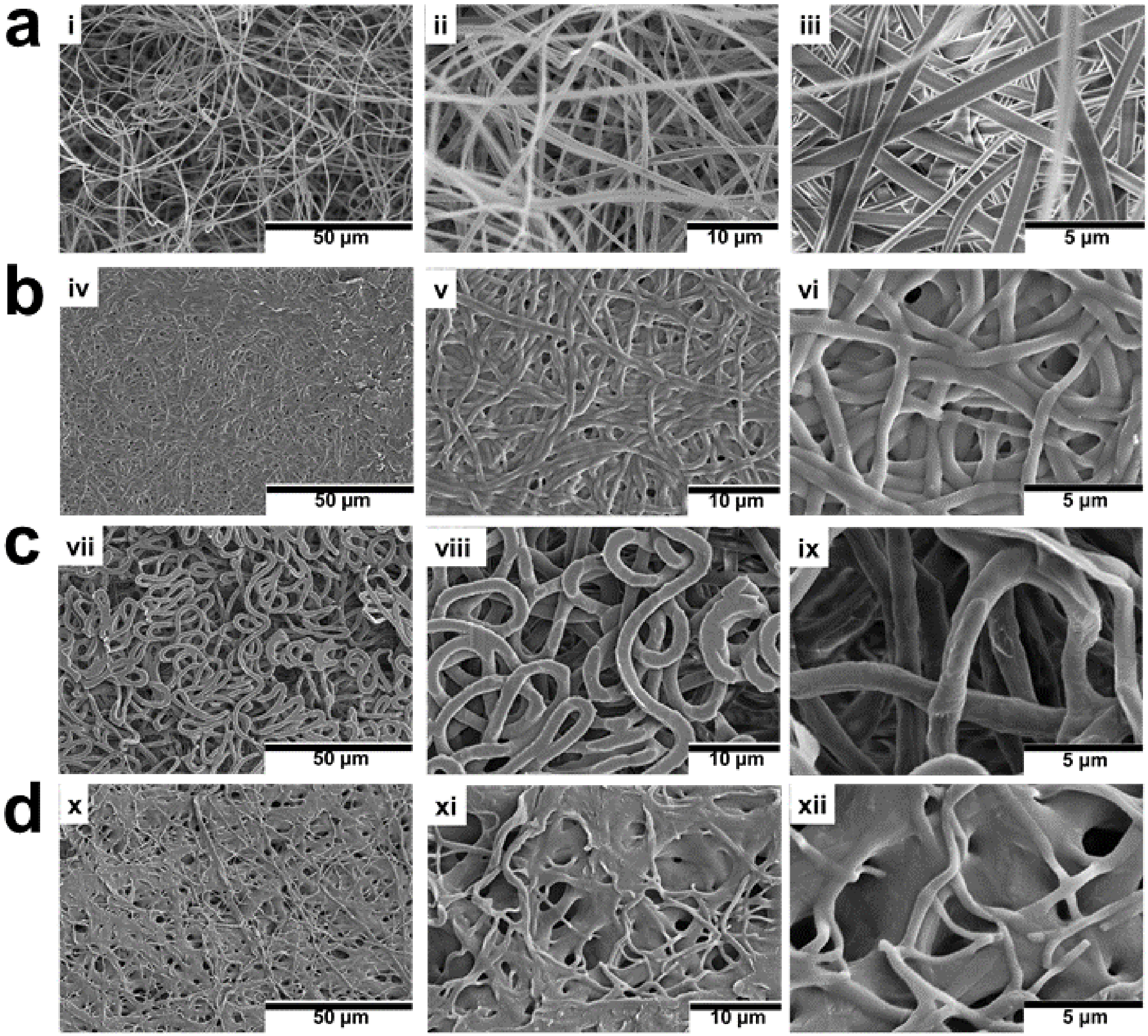

Supplementary Fig. 2 The morphology of the electrospun fibers scaffolds from the scanning electron microscopy (SEM) analysis with various magnifications of **a** GelMA **b** GelMA-UV **c** PCL and **d** PCLMA.

Supplementary Tables

Supplementary Table 1. Contact angle measurements and surface energy of the fibers. Each mean value corresponds to the average value on three different samples.

Sample	Contact angle (°)		Surface free energy (mN m^{-1})			$\frac{\gamma_p}{(\gamma_d + \gamma_p)}$
	**Water**	**Diiodomethane**	**Dispersive (γ_d)**	**Polar (γ_p)**	**Total**	
**PCLMA-UV**	81 ± 3.3	43 ± 1.0	38.3	3.5	41.7	0.08
**PCLMA:GelMA (70:30)**	105 ± 2.0	24 ± 0.9	46.6	0.43	47.1	0.01
**PCLMA:GelMA (70:30)-UV**	88 ± 3.3	30 ± 1.9	44.3	0.89	45.2	0.02

Supplementary Table 2. Histological grading scale for soft tissues.

Histological grading of:	Explanation	Grading
**Capsule thickness**	1–4 cell layers	4
	5–9 cell layers	3
	10–30 cell layers	2
	>30 cell layers	1
	Not applicable	0
**Tissue response of the capsule surround implants**	Fibrous, mature, not dense, resembling connective or fat tissue in the non-injured regions	4
	Fibrous, but immature, showing fibroblasts and little collagen	3
	Granules and dense, containing both fibroblasts and many inflammatory cells	2
	Consists of masses of inflammatory cells with little or no signs of connective tissue organization	1

	Cannot be evaluated because of infection or factors not necessarily related to the material	0
**Tissue response directly adjacent to the implant surface (interface)**	Fibroblasts contact the implant surface without the presence of macrophages or foreign body giant cells	4
	Scattered foci of macrophages and foreign body cells are present	3
	One layer of macrophages and foreign body cells is present and multiple layers of macrophages and foreign body cells are present	2
	Multiple layers of macrophages and foreign body cells are present	1
	Cannot be evaluated because of infection or other factors not necessarily related to the material	0
**Blood vessel**	Intense	3
	Moderate	2
	Minimal	1
	Absent	0

Supplementary Notes

Supplementary Notes 1: Procedure for the preparation of electrospun fibers

The fibers were prepared by electrospinning a solution of respective polymer (10 wt.%) or polymer mixture (Fig. 3a) in vials (10 mL) containing HFIP. Subsequently, the vials were closed and dissolved under stirring at room temperature for 24 h. Firstly, the solutions were placed in a syringe (BD Yale, 3 mL) which was then affixed with a needle (Inbras, 23G). The electrospinning was carried out using 17 kV (Nanospinner Machine, Inovenso) as the positive voltage. An aluminum foil collector plate (20 cm x 20 cm square plate) was selected as the anode, a needle-collector distance of 10 cm was used, and a solution flow rate of 1 mL/h was applied (Harvard, PHD 2000). The temperature and humidity were controlled at 21–23 °C and 44–54%, respectively.

Supplementary Notes 2: Procedure for the preparation of PCLMA

The electrospun PCL fibers were plasma treated (100 W) for 5 min and subsequent, immersed in 3-(Trimethoxysilyl)propyl methacrylate in a glass container for 24 h at room temperature. Next, the material was washed several times with water and centrifuged to remove unreacted silane and then further dried under vacuum, affording silane methacrylated PCL (PCLMA).

Supplementary Notes 3: General procedure for the preparation of GelMA.[1]

10 g of gelatin type A from porcine skin was dissolved in PBS (100 mL) at 50 ºC. The mixture was agitated and stirred vigorously until a homogenous solution was obtained (~1 h). Subsequently, 3 mL of methacrylate anhydride was added dropwise to the reaction vessel, and the resulting mixture was kept stirring for 3 h at 50 ºC. Afterwards, the mixture was diluted with preheated PBS (50 ºC), and stirred for 10 min at the stated temperature. Then, the mixture was dialyzed against (DI) water using dialysis tubing (12-14 kDa MWCO, Spectrum Lab Inc.) for 7 days at 40 ºC. During the time, the water was changed at least once a day. After dialysis, the resulting clear solution was lyophilized affording GelMA as a white solid (~71% yield). The degree of methacryloyl substitution was detetermined using ^{1}H NMR analysis.

Supplementary Notes 4: Wettability analysis

Before analysis, a careful calibration was performed in accordance to the equipment manual and all the scaffolds were fixed on a Teflon base. Then, DI water (2 µL) and diiodomethane (2 µL) were dropped onto the scaffold surfaces and measured after 1 s. The atmosphere and humidity (~ 60%) were controlled during all the measurements. The surface energy was calculated as described in the Supplementary Notes 9.

Supplementary Notes 5: Mechanical experiments

The samples were prepared as depicted in Fig 3b. Rectangular specimens (dimensions 2.5 cm x 0.5 cm x 0.1 cm) were cut and glued to paper and prior to the measurements, were cut at the edge. A tensile test was performed immediately with a strain rate of 10 mm/min until failure, and the ultimate tensile stress was obtained at the failure point, while the elastic modulus was determined from the slope of the stress-strain curve. The elongation-at-break (%) was also determined. For each material, at least three repetitions were performed and the values are reported as mean ± SD, N = 3. ANOVA ($p < 0.05$) followed by the Tukey's multiple comparisons test was used for determining statistical significance.

Supplementary Notes 6: ^{1}H NMR analysis

For the ^{1}H-NMR analysis, the solvent resonance resulting from incomplete deuterium incorporation was employed as the internal standard ($CDCl_3$ = 7.26 ppm). The data were processed using TopSpin 3.6.1 software (Bruker, Harvard, MA) by comparing the respective starting material's chemical shifts. The degree of substitution (methacrylation) was determined by comparing the peaks of the methacrylate protons at 6.04 ppm (1H) and 5.48 ppm (1H) and the protons of PCL at 2.29 ppm (2H). Based on this comparison, the degree of substitution of PCL providing PCLMA was determined to be 61% by NMR analysis (Figs. 2b, S1).

Supplementary Notes 7: ^{1}H NMR analysis for the quantification of physical and covalent bonding

The degree of covalent bonding on the fibers were determined by dissolving the PCLMA fibers in dichloromethane and then reprecipitating in cold methanol. The procedure was repeated three times to ensure that all of the non-covalently bonded silane was removed. The solid material was then dried under vacuum prior to NMR analysis.

Supplementary Notes 8: Thermal behavior of the fibers

The thermal behavior of the samples was analyzed by taking 5 mg of the fibers for the experiments using an aluminum pan, temperature ranges between –20 to 200 ºC, at a heating rate of 5 ºC/min. The crystallinity was calculated following equation 1:

$$Xc\ (\%) = \frac{\Delta Hm}{\Delta Hm^{\infty} x\ W}\ x\ 100 \qquad (1)$$

Where: ΔHm = melting enthalpy of the sample (J/g) and ΔHm^{∞} = melting enthalpy of a 100% crystalline sample (for 100% crystalline PCL ΔHm = 136 J/g). W is the mass fraction of PCL in the sample.[2]

Supplementary Notes 9: Contact angle and surface energy measurements

The experiments were performed by taking 2 µL of each liquid (water and diiodomethane) and dropped on each scaffold and images were recorded after 1 min. N = 3 for each sample was used. Contact angle measurements were performed in triplicate in a Phoenix 150 contact angle system, using water and

diiodomethane as the liquid. The contact angle (θ) was measured using the software ImageJ, and the values were used to calculate the surface free energy (γ) of the materials, as well its dispersive (γ^d) and polar (γ^p) parts by using the Owens-Wendt equation (2):[3]

$$1 + \cos\theta = 2\sqrt{\gamma_s^d}\left(\frac{\sqrt{\gamma_l^d}}{\gamma_l}\right) + 2\sqrt{\gamma_s^p}\left(\frac{\sqrt{\gamma_l^p}}{\gamma_l}\right) \quad (2)$$

θ - contact angle measured between the solid sample and the liquid (diiodomethane or water)

γ_s^d - dispersive component of surface free energy of the examined materials

γ_s^p- polar component of surface free energy of the examined materials

γ_l - surface free energy of the liquid (diiodomethane equal to 50.8 mJ/m^2 or water equal to 72.8 mJ/m^2)

γ_l^d- dispersive component of the liquid (diiodomethane equal to 50.8 mJ/m^2 or water equal to 21.8 mJ/m^2)

Supplementary Notes 10: *In vivo* biocompatibility study

All of the animal handling and surgical procedures were strictly conducted according to the Guiding Principles for the Use of Laboratory Animals. This study was approved by the Animal Care Committee guidelines of the São Carlos Federal University (protocol 8577280716). 10 male Wistar rats weighing 210–260 g and aged 8 weeks were used. During the experimental period, the animals were maintained under controlled conditions of light–dark periods of 12 h and temperature (24 ± 2 ºC), with free access to water and commercial diet. Initially, the animals were anesthetized with Ketamine (80 mg/kg) and Xylazine (10 mg/kg). To insert the subcutaneous implants, rats were immobilized on their dorsal region, and the skin was shaved and disinfected with iodine. In each animal, four incisions of approximately 8 mm were made along the back, 2 being on the left and 2 on the right side, using sterile fields, followed by divulsion with straight surgical scissors. The implants were randomly placed and the skin was sutured with a 4-0 nylon monofilament suture (ShalonVR). The animals were housed in pairs. In the initial postoperative period, the intake of water and food was monitored. Furthermore, the animals were observed for signs of pain, infection, and proper activity. Animals were euthanized after 5 days with a lethal dose of anesthetic (Ketamine/Xylazine) and the biomaterials were harvested with surrounding tissue for histopathological analysis.

Supplementary Notes 11: Histopathological analysis

The specimens were fixed in 10% buffered formalin (Merck, Darmstadt, Germany) for 24 h, followed by dehydration in a graded series of ethanol and embedding in paraffin. In the transverse axis to the implant, thin sections (5 μm) were prepared using a microtome (Leica Microsystems SP 1600, Nussloch, Germany).

The specimens were stained with hematoxylin and eosin (H.E. stain, Merck) and examined using optical microscopy (Olympus Optical Co., Tokyo, Japan). The tissue response to subcutaneous implants was analyzed semi-quantitatively, and a histological grading scale[4-6] was used to evaluate the capsule thickness, the tissue response of the capsule surrounding the subcutaneous implant, and also the tissue directly adjacent to the implant surface. These evaluations were performed in four predetermined fields of at least two sections of each specimen. Two experienced observers performed the scoring (Table S2) in a blinded manner.

Supplementary Notes 12: *In vivo* hernia application

Three B6/CBA F1 mice, strain 8 weeks old from the Multidisciplinary Center for Biological Research, were employed for each group for the *in vivo* analysis of the various nanofibers. The protocol followed the guidance of Ethical Committee for Laboratory Research Use of University of Campinas in SP- Brazil, which approved the procedures. The animals were anesthetized with 100 mg/Kg ketamine and 10 mg/Kg xylazine IP before being submitted to surgery for the acquisition of a ventral abdominal hernia model.[7] and adapted for mice analysis. Hernias were subsequently repaired with the various PCL-based nanofibers. Immediately after the surgery, as well as three days following, a 5 mg/Kg single dose of ketoprofen for analgesic and anti-inflammatory effects was administrated. After 29 days, the animals were euthanized by overdosing with isoflurane, and the implanted material, as well as the surrounding tissue, were harvested. After 24 h of soaking the samples in formaldehyde 10%, the materials were processed for histological analysis. Hematoxylin and Eosin (H&E) were employed for H&E staining, and solutions of Hematoxylin, Acid Fuchsin and Methyl Blue were prepared for the Masson's Trichrome staining to distinguish the collagen fibers. Subsequently, tissue sections (5 μm) were placed on slides and observed under regular light microscopy. For obtaining stretch fibers images, following the H&E staining samples were further studied under microscopy with a fluorescence filter (590–630 nm), and elastic fibers were detected by eosin fluorescence as suggested by Heo et al.[8] For all samples, 300 ms of fluorescence acquisition time was used to standardize the exposition time for all slides and images.

Supplementary Notes 13: General observation of the experimental animals

No animals died during the experimental period. The rats and mice showed no postoperative complications, and they quickly returned to their normal diet and showed no loss of body mass. Furthermore, no infections in the injured areas were detected.

Supplementary Notes 14: Quantitative matters of connective tissue

For quantitative data analysis, a double-blind analysis was performed, i.e. the analyzer did not have data for features of the different tested nanofibers for biological analysis. ImageJ software was employed, regarding the quantification of the amount and density of the fibers of connective tissue found on the tissue adjacent to the materials, in the interface between the nanofibers and the tissue surrounding the materials. We collected images to quantify the total collagen content using regular light microscopy on the slides treated with Masson's trichrome staining method, and for stretch fiber quantification, we collected images, using fluorescent microscopy, of slides stained with the H&E staining method. The images were processed using ImageJ software, and to determine the total collagen, the histogram tool of the software was employed on live mode and standardized with the measurements in the blue channel by adjusting the threshold color: 0–185 Hue; 0–255 Saturation, and 0–135 Brightness. To procure an accurate measurement, the threshold color was processed in white for all the images that were analyzed. To further evaluate the number of the stretch fibers from fluorescence images, the mean gray value was measured. The data were collected by limiting individual analyses to circular areas having diameters of 100 μm, and multiples spots from the same image were evaluated. The statistical analysis was performed using ANOVA by Kruskal-Wallis test with multiple comparisons in GraphPad Prism 6 software. A scoring table with subjective data was derived to appraise biological parameters observed using histological analysis. Specifically, immunological cells, amounts, and *in vivo* distributions; tissue recovery and appearance in the regions surrounding the implants; and blood vessel formation were investigated.

Supplementary Notes 15: Antibacterial study-colony count assays

The antibacterial activity of the electrospun fibers was tested on *Staphylococcus aureus* (*S. aureus*; ATCC 25923)*, Methicillin-resistant S. aureus* (MRSA) (ATCC 43300), and *Pseudomonas aeruginosa* (*P. aeruginosa*; ATCC 25668). The bacteria were inoculated in 4 mL of 3% tryptic soy broth (TSB) and cultured overnight, for 12 hours, in a shaker incubator operating at 130 rpm and at 37 ºC. On the following day, optical density readings using a SpectraMax (M3) plate reader enabled the quantification of the bacterial concentrations after culture. Specifically, readings were obtained at the absorbance wavelength, λ_a, equal to 562 nm, and bacterial dilutions were prepared until optical density outputs converged to 0.52, which indicated bacterial concentrations of 10^9 CFU/mL. Bacterial suspensions were consequently diluted 100,000x in TSB, to a cell seeding concentration of 10^4 CFU/mL. The electrospun substrates (10 mm x 10 mm x 0.5 mm), which included PCL, PCLMA, GelMA-UV, PCLMA:GelMA (70:30) and PCLMA:GelMA

(70:30)-UV, were distributed into the wells of 24-well polystyrene plates, in triplicate for each bacterial type, and inoculated each with 1 mL of the 10^4 CFU/mL bacterial suspensions. The 24-well plates containing the treated test substrates were placed in a stationary incubator under standard conditions (humidified, 5% CO_2, 37 ºC) for 23 hours. After 23 hours, the samples were washed three times gently with phosphate buffered saline (PBS, pH ~7.4) and transferred to sterile 24-well plates. The test substrates were subsequently deposited into Eppendorf tubes containing 1 mL of PBS and vortexed continuously for 15 min, after which dilutions of the test suspensions were serially prepared (10x; 100x; 1000x; 10000x; 100000x) and dropped onto TSA plates in 10 µL aliquots. The TSA plates were air dried in a sterile environment, covered, and inverted before being placed inside a stationary incubator (humidified, 5% CO_2, 37 ºC). Following the interim period of 15 hours, plates were removed from the incubator and bacterial colonies were counted manually.

Supplementary Notes 16: Detection of reactive oxygen species (ROS) production

Bacteria were cultured and samples inoculated as in the microbial colony counting procedure described above. Briefly, 1 mL of 10^4 CFU/mL of bacterial suspensions were deposited onto fibers inside 24-well plates. After 23 hours of incubation at 37 ºC in a humidified, 5% CO_2 atmosphere, the fibers were transferred into sterile 24-well plates and washed with PBS three times gently. The H_2DCFDA was constituted in dimethyl sulfoxide (DMSO) to a final concentration of 10 mM, and a 10 mM staining solution was subsequently prepared by diluting the constituted H_2DCFDA in TSB. 1 mL of the staining solution was used to completely immerse each of the test substrates. Co-incubation of the substrates and the ROS detector was carried out in the absence of light for 30 min at 5% CO_2 and 37 ºC, after which the staining solution was discarded and replaced by PBS. The fibers were carefully transferred to Eppendorf tubes containing 1 mL of PBS and vortexed for 5 min to detach colonizing bacteria. The emanant suspensions were distributed into the wells of sterile 24-well plates, and spectrophotometric measurements of the samples were taken, with excitation and emission wavelengths $\lambda_{ex/em}$ ~492/517 nm, consistent with those for detecting dichlorofluorescein (DCF) fluorescence.

Supplementary Notes 17: Scanning electron microscopy (SEM) analysis for bacterial adhesion

S. aureus, *MRSA*, and *P. aeruginosa* were inoculated into TSB and cultured overnight, as described in Supplementary note 15, before being quantified and deposited (1 mL) onto the test substrates, at the initial

concentration of 10^4 CFU/mL. After 23 hours of exposure of the substrates to the bacteria, the samples were transferred to a sterile 24-well plate and washed three times lightly with PBS. After complete aspiration of the final wash of PBS solution, the adherent cells were cross-linked using 2.5% of glutaraldehyde dispersed in 0.1 M cacodylate buffer as the primary fixative. The fibers were completely immersed in 1 mL of a glutaraldehyde solution, and the 24-well plates were maintained at 4 ^{o}C for 1 week before being dehydrated serially in graded ethanol. The fibers were exposed once to each of 30%, 50%, 70%, 80%, and 90% ethanol and three times consecutively to 100% ethanol, with each immersion being sustained over the span of 15 min. The final dehydration step was immediately succeeded by critical point drying of the samples in CO_2, using a Samdri-PVT-3D (Tousimis Research Corporation; Rockville, MD) manual critical point dryer. When the samples were completely depleted of moisture, they were affixed to aluminum mounts and coated with a 5-nm thick layer of platinum using an automatic sputtering device. The mounts were subsequently loaded into a Hitachi S-4800 SEM for imaging.

References

1. Nichol, J. W. *et al.* Cell-laden microengineered gelatin methacrylate hydrogels. *Biomaterials* **31**, 5536–5544 (2010).
2. Pitt, C. G., Chasalow, F. I., Hibionada, Y. M., Klimas, D. M. & Schindler, A. Aliphatic polyesters. I. The degradation of poly(ϵ-caprolactone) in vivo. *J. Appl. Polym. Sci.* **26**, 3779–3787 (1981).
3. Owens, D. K. & Wendt, R. C. Estimation of the surface free energy of polymers. *J. Appl. Polym. Sci.* **13**, 1741–1747 (1969).
4. Jansen, J. A., Dhert, W. J. A., Van Der Waerden, J. P. C. M. & Von Recum, A. F. Semi-Quantitative and Qualitative Histologic Analysis Method for the Evaluation of Implant Biocompatibility. *J. Invest. Surg.* **7**, 123–134 (1994).
5. Renno, A. C. M. *et al.* Incorporation of bioactive glass in calcium phosphate cement: An evaluation. *Acta Biomater.* **9**, 5728–5739 (2013).
6. Link, D. P. *et al.* Evaluation of the biocompatibility of calcium phosphate cement/PLGA Microparticle composites. *J. Biomed. Mater. Res., Part A* **87A**, 760–769 (2008).
7. Suckow, M. A., Boynton, F. D. D. & Johnson, C. Use of a Rat Model to Study Ventral Abdominal Hernia Repair. *J. Vis. Exp.* **128**, e53587 (2017).
8. Heo, Y. S. & Song, H. J. Characterizing cutaneous elastic fibers by eosin fluorescence detected by fluorescence microscopy. *Ann. Dermatol.* **23**, 44–52 (2011).
