# Supplementary material for: Engineering multifunctional bactericidal nanofibers for abdominal hernia repair
Source: Commun Biol. 2021 Feb 19;4:233. doi: 10.1038/s42003-021-01758-2 (PMC7896057; doi:10.1038/s42003-021-01758-2)
Supplement: Supplementary file 3 — Description of Additional Supplementary Files [file 42003_2021_1758_MOESM3_ESM.pdf]

Description of Additional Supplementary Files

Name: **Supplementary Movie 1.**

Description: Demonstrating the poor stability of the GelMA fiber in aqueous solution (PBS), which immediately dissolved upon contact with the PBS.

Name: **Supplementary Data 1**

Description: **Sheet (1)** Raw Data for figure 2b the ^{1}H NMR spectra. **Sheet (2)** Raw Data for figure 2e the FTIR spectra.

Name: **Supplementary Data 2**

Description: **Sheet (1)** Raw Data for figures 3d and 3e the mechanical testing.

Name: **Supplementary Data 3**

Description: **Sheet (1)** Raw Data for figure 4a the DSC thermograms. **Sheet (2)** Raw Data for figure 4c the contact angle. **Sheet (3)** Raw Data for figure 4d the light transmittance analysis. **Sheet (4)** Raw Data for figure 4f the fiber diameter.

Name: **Supplementary Data 4**

Description: **Sheet (1)** Raw Data for figure 5a the *in vitro* degradation. **Sheet (2)** Raw Data for figure 5b the cell viability. **Sheet (3)** Raw Data for figure 5d the histological grading scale.

Name: **Supplementary Data 5**

Description: **Sheet (1)** Raw Data for figure 6b the quantification of connective tissue.

Name: **Supplementary Data 6**

Description: **Sheet (1)** Raw Data for figures 7a and 7b the bactericidal results.
