# Supplementary material for: Engineering multifunctional bactericidal nanofibers for abdominal hernia repair
Source: Commun Biol. 2021 Feb 19;4:233. doi: 10.1038/s42003-021-01758-2 (PMC7896057; doi:10.1038/s42003-021-01758-2)
Supplement: Supplementary file 11 — Reporting Summary [file 42003_2021_1758_MOESM11_ESM.pdf]

nature research

Corresponding author(s): Anderson Oliveira Lobo

Last updated by author(s): 10/16/2020

Reporting Summary

Nature Research wishes to improve the reproducibility of the work that we publish. This form provides structure for consistency and transparency in reporting. For further information on Nature Research policies, see our Editorial Policies and the Editorial Policy Checklist.

Statistics

For all statistical analyses, confirm that the following items are present in the figure legend, table legend, main text, or Methods section.

n/a	Confirmed	
[ ]	[x]	The exact sample size (n) for each experimental group/condition, given as a discrete number and unit of measurement
[ ]	[x]	A statement on whether measurements were taken from distinct samples or whether the same sample was measured repeatedly
[ ]	[x]	The statistical test(s) used AND whether they are one- or two-sided<br>*Only common tests should be described solely by name; describe more complex techniques in the Methods section.*
[ ]	[x]	A description of all covariates tested
[x]	[ ]	A description of any assumptions or corrections, such as tests of normality and adjustment for multiple comparisons
[ ]	[x]	A full description of the statistical parameters including central tendency (e.g. means) or other basic estimates (e.g. regression coefficient) AND variation (e.g. standard deviation) or associated estimates of uncertainty (e.g. confidence intervals)
[ ]	[x]	For null hypothesis testing, the test statistic (e.g. F, t, r) with confidence intervals, effect sizes, degrees of freedom and P value noted<br>*Give P values as exact values whenever suitable.*
[x]	[ ]	For Bayesian analysis, information on the choice of priors and Markov chain Monte Carlo settings
[x]	[ ]	For hierarchical and complex designs, identification of the appropriate level for tests and full reporting of outcomes
[x]	[ ]	Estimates of effect sizes (e.g. Cohen's d, Pearson's r), indicating how they were calculated

Our web collection on statistics for biologists contains articles on many of the points above.

Software and code

Policy information about availability of computer code

Data collection	n/a
Data analysis	GraphPad Prism 6 software

For manuscripts utilizing custom algorithms or software that are central to the research but not yet described in published literature, software must be made available to editors and reviewers. We strongly encourage code deposition in a community repository (e.g. GitHub). See the Nature Research guidelines for submitting code & software for further information.

Data

Policy information about availability of data

All manuscripts must include a data availability statement. This statement should provide the following information, where applicable:

- Accession codes, unique identifiers, or web links for publicly available datasets
- A list of figures that have associated raw data
- A description of any restrictions on data availability

Data available on request from the authors.

Field-specific reporting

Life sciences study design

All studies must disclose on these points even when the disclosure is negative.

Sample size	We used 3 animals for each group. Each animal had five histological slides with three tissue cuts per slide. Number of animals was estimated in accordance of ethical criteria from the Ethical Committee using a statistical calculation from simple group analysis considering the reliability estimate (Log β) and sample error estimate (Log p), in which we got a number of 3 animals per group.<br>The formula used was: n = (Log β) / (Log p) -> n = log 0.05 / log 0.33 = 2.70 ~ 3 animals<br>Reference - Dell RB, Holleran S, Ramakrishnan R. Sample Size Determination. Ilar Journal. 2002; 43 (4): 207-213.
Data exclusions	In the histological analysis we unconsidered the healthy tissues (muscle and fat tissues not disturbed) and considered for counting analysis the tissue in regeneration containing fibers of collagen in ongoing recover.
Replication	we are working with live animals; each animal was considered replication since the results were quite similar among the samples in the same group, representing good replicability.
Randomization	Each group was kept in a different cages after surgery, and each group was named with letters as "Group A"; "Group B" etc. by the technician who took care of animals once a week without any clue about the specificity of the research, ordered to take notes if something weird or wrong happened, which did not happened.
Blinding	We made the experiment keeping blindness among the researchers responsible for the surgery and the final analysis; the technician who took care of the animals and who named the groups and another technician who made the slides with the cuts for histological analysis. Only after histological analysis of groups A, B etc., the researchers and the technicians gathered and analyzed the material used corresponded for each group. Then they discussed the results.

Reporting for specific materials, systems and methods

We require information from authors about some types of materials, experimental systems and methods used in many studies. Here, indicate whether each material, system or method listed is relevant to your study. If you are not sure if a list item applies to your research, read the appropriate section before selecting a response.

Materials & experimental systems

n/a	Involved in the study	
☒	☐	Antibodies
☒	☐	Eukaryotic cell lines
☒	☐	Palaeontology and archaeology
☐	☒	Animals and other organisms
☒	☐	Human research participants
☒	☐	Clinical data
☒	☐	Dual use research of concern

Methods

n/a	Involved in the study	
☒	☐	ChIP-seq
☒	☐	Flow cytometry
☒	☐	MRI-based neuroimaging

Animals and other organisms

Policy information about studies involving animals; ARRIVE guidelines recommended for reporting animal research

Laboratory animals	Surgical procedures were conducted according to the Guiding Principles for the Use of Laboratory Animals. This study was approved by the Animal Care Committee guidelines of the São Carlos Federal University (protocol 8577280716). 10 male Wistar rats weighing 210–260 g and aged 8 weeks were used.<br>Three B6/CBA F1 mice, strain 8 weeks old from the Multidisciplinary Center for Biological Research, were employed for each group for the in vivo analysis of the various nanofibers. The protocol followed the guidance of Ethical Committee for Laboratory Research Use of University of Campinas in SP- Brazil, which approved the procedures.
Wild animals	The study did not involve any wild animals
Field-collected samples	The study did not involve sample collection from the field
Ethics oversight	The study was approved by the Animal Care Committee guidelines of the São Carlos Federal University (protocol 8577280716) and by Ethical Committee for Laboratory Research Use of University of Campinas in SP- Brazil,

Note that full information on the approval of the study protocol must also be provided in the manuscript.
